# Supplementary material for: Diverse Sphingolipid Species Harbor Different Effects on Ire1 Clustering
Source: Int J Mol Sci. 2022 Oct 12;23(20):12130. doi: 10.3390/ijms232012130 (PMC9602660; doi:10.3390/ijms232012130)
Supplement: Supplementary file 1 [file ijms-23-12130-s001.zip › ijms-1942835-supplementary.pdf]

Supplemental Table S1. Yeast strains used in this study

Strain	Genotype	Reference
CTY182	*MATa ura3-52 lys2-801 Dhis3-200*	45
CTY5-2	*MATa ura3-52 his3-200 trp1Δ lys2-801 sec14-3*	45
CTY252	*MATα ura3-52 sec12-4*	45
CTY1920	*Mat a, sec14-1, tlg2Δ::KanMX4, ade2-101 his3Δ-1, leu2Δ, ura3Δ*	30
CTY1958	*Mat a, sec14-1, kes1-1, ura3-52, his3-200, lys2-801, tlg2Δ::KanMX4*	30
BY4742	*MATα his3Δ1 leu2Δ0 lys2Δ0 ura3Δ0*	46
SSS1	*MATα ade2- 1 ura3-1 his3-11,15 trp1-1 leu2-3,112 can1-100 sss1Δ::KanMX4* pJKB2	40
sss1-6	*MATα ade2- 1 ura3-1 his3-11,15 trp1-1 leu2-3,112 can1-100 sss1Δ::KanMX4* pJKB16	40
sss1-7	*MATα ade2- 1 ura3-1 his3-11,15 trp1-1 leu2-3,112 can1-100 sss1Δ::KanMX4* pCM205	40

Supplemental Table S2. Plasmids used in this study

Plasmid	Genotype	Reference
pMS383	Ire1-mCherry expression plasmid	31
pRS313	YCp *HIS3*	47
pJKB2	YCp *SSS1 HIS3*	40
pJKB16	YCp $sss1^{P74A\ I75A}$ *HIS3*	40
pCM205	YCp $sss1^{H72K}$ *HIS3*	40
pUH7	*URA3* → *HIS3* marker swap plasmid	43
pCM220	YCp (P_{DOX}-*KES1, HIS3*)	This study
pCM221	YCp (P_{DOX}-$KES1^{Y97F}$, *HIS3*)	This study
pCM222	YCp (P_{DOX}- $KES1^{K109A}$, *HIS3*)	This study
